# Attitude and willingness of biodiversity conservation in Chinese university students: Associated factors and the mediation of social support

**DOI:** 10.1371/journal.pone.0307510

**Published:** 2024-07-19

**Authors:** Yuan Zhang, Mo-Lin Hou, Chang-Qi Chen, Zhou-Yang Liao, Yun-Fang Guan, Yu-Lin Yuan, Yin Zhang, Min-Yan Zhao, Tian-Tian Tang

**Affiliations:** 1 College of Biodiversity Conservation, Southwest Forestry University, Kunming, China; 2 Institute of Tibetan Plateau Research, Chinese Academy of Sciences, Beijing, China; Tianjin University, CHINA

## Abstract

In this cross-sectional study of 1475 Chinese university students, we explored associated factors of attitude and willingness of biodiversity conservation, analyzed the hypothesized mediation by social support in the association between attitude and willingness of biodiversity conservation. Multivariate logistic regression model revealed that major and social support were prominently related to both attitude and willingness of biodiversity conservation. Besides, path model identified a statistically significant mediation by social support, sex, race, and family residence presented noticeable effect modification on the mediation of social support. These major findings suggest that intervention measures which aiming at enhancing social support could be considered for elevating attitude and willingness of biodiversity conservation among Chinese university students.

## 1. Introduction

Biodiversity is essential for the living of all life on earth, including humans. Nevertheless, climate change and human activities have been contributing to expedited loss of biological diversity [1]. Therefore, effective conservation of biodiversity is critical for human survival. Younger generations are main practitioners for future sustainable development, education in biodiversity conservation is important in shaping young people’s attitudes toward biodiversity conservation [2]. Although in the past three decades, tremendous efforts have been made by the Chinese government in ecological protection and environmental management [3], compared with many students in Western countries, a lack of formal education regarding environmental education in Chinese schools has been identified as an issue [4].

The Knowledge, Attitude and Practice (KAP) theory illustrates that the process of human behavior change can be divided into three phases: acquiring knowledge, generating attitudes, and performing practice [5]. After been proposed, the KAP theory has been widely used, especially in the field of health-related behaviors [6]. For instance, some researchers used the KAP model to understand community behaviors which related to COVID-19 pandemic [7]. In recent years, the KAP theory has also been proved highly applicable to environmental conservation and management studies [8]. However, for Chinese university students, although the KAP theory had been adopted in investigating marine environment pollution, ecotourism, and pro-environmental behavior [9–11], no published studies have ever thoroughly discussed biodiversity conservation of Chinese university students by using the KAP model.

Among the three stages covered by the KAP theory, knowledge acquiring is comparatively easier, therefore, finding associated factors of attitude shaping and practice implementing are crucial. Social support is an important concept in the field of positive psychology. It refers to the perception and actuality that one has cared for, has assistance from other people [12]. In a medical study published in 2016, the authors found that social support significantly influenced the association between attitude and practice in medical decision making [13], another Indonesian study suggested that perceived social support probably moderated the relationship between the attitude of becoming a social entrepreneur and social entrepreneurial intention [14]. Therefore, it is likely that perceived social support may play a role in the association between attitude and practice of biodiversity conservation, however, this hypothesis has not been tested.

In the current study, aiming to address the deficiencies in the field, by surveying a large sample of Chinese university students, we intend to explore associated factors of attitude and willingness to practice in biodiversity conservation. Moreover, we analyzed the possible mediation of perceived social support in the association between biodiversity attitude and willingness to practice.

## 2. Methodology

### 2.1 Study design

A cross-sectional survey was conducted in February 2023 in Southwest Forestry University, Yunnan Province, China. We adopted a stratified random clustering sampling method which proportionate to sample size to choose study subjects: all registered students in this university were firstly stratified into different layers by their affiliated school, grade, and major; then, within each layer, according to precalculated proportion in the final sample size, several clusters (classes of students) were randomly chosen. We used formula for simple random sampling to calculate for preliminary required sample size: as no previously published studies have reported estimated prevalence of attitude or practice for biodiversity conservation in college students, we set it as the most conservative 50%, statistical significance set as 0.05, acceptable error set was no more than 15%, we yielded a sample size of 1112. Considering that the sampling error of simple clustering sampling will be inevitably larger than simple random sampling, we further adopted a design effect of 1.2 to adjust for the calculated sample size, and the final reached required sample size was 1335.

### 2.2 Data collection

We used a self-developed questionnaire to collect relevant information from the survey participants. This questionnaire is a comprehensive tool comprised of multiple modules, measuring demographics, social support, biodiversity conservation attitude and willing to practice, environmental protection awareness, etc. All questionnaires were self-administered by the respondents, who had been gathered in patches in a classroom, and sat separately, upon completion, quality control personnels deployed at site will check for completeness and quality of filled questionnaires. The Epidata (Version 4.2) software was used for data management, double entry was performed to guarantee data inputting quality. Prior to survey, a pre-survey of 120 students was done to check for performance of the instrument, and the results revealed ideal internal validity and test-retest reliability, with the Cronbach’s α for all modules ranged from 0.87 to 0.94, and the Pearson’s correlation coefficients were all above 0.85 (0.89–0.92). The study protocol was reviewed and approved by the Ethics Committee of Southwest Forestry University. Written informed consents were obtained from all respondents prior to the survey.

### 2.3 Variables and definitions

#### Attitude and willingness to practice biodiversity conservation

In the self-developed questionnaire, attitude of biodiversity conservation was measured by a single question stated as “How do you think the importance of biodiversity conservation to human’s life and work?”, with 5-point Likert style responses (not important, somewhat important, important, very important, extremely important); willingness to practice biodiversity conservation was reflected by another question stated as “At what extent you are willing to participate in biodiversity conservation activities that around you?”, also with 5-point Likert responses (very much unwilling to, unwilling to, hard to say, willing to, very much willing to).

#### Perceived social support

Considering that for university students, their major sources of social support are similar to junior or senior high school students, therefore we used the Chinese version of Child and Adolescent Social Support Scale (CASSS) to gauge perceived social support. The CASSS contains 40 questions which can be divided into 4 dimensions (10 questions for each dimension), measuring social support from parents, teachers, classmates, and close friends, respectively. Responses to every question in the CASSS are 5-point Likert style, measuring frequencies from “Never” to “Always” [15]. A higher combined score of the CASSS indicates higher level of perceived social support.

#### Statistical analysis

Descriptive statistics together with suitable statistical tests were used to depict characteristics and compare differences between subgroups. Univariate and multivariate binary logistic regression models were used to explore associated factors of attitude and willingness to practice of biodiversity conservation. As attitude and willingness to practice were all ordinal variables originally, before performing analysis, we dichotomized them accordingly: for attitude, we intended to explore the associated factors of high-level perceived importance in biodiversity conservation, therefore the answers were recoded as “very important” (those who answered “very important” or “extremely important”) and “not very important” (those who answered otherwise); for willingness to practice, we intended to screen for associated factors of confirmative willingness in practicing biodiversity conservation, therefore the answers were recoded as “willing to” (those who answered “willing to” or “very much willing to”) and “not willing to” (those who answered otherwise). The hypothesized mediation of social support was analyzed using the path analysis. A series of subgroup analysis were further performed to check for possible effect modification by key characteristics in the mediation of social support. All statistical analyses were executed using the R software (Version 4.2.2). Except for univariate logistic regression models, which adopted a comparatively loose criterion (*p*<0.10), the statistical significance for all the rest analyses was set as a two-tailed *p* value less than 0.05.

## 3 Results

### 3.1 General characteristics of study subjects

After initial sampling procedure, we successfully recruited 1623 students, 148 of them were excluded because of invalid questionnaire (incomplete or illogical), therefore the final analysis was based on a total sample size of 1475, with a valid response rate of 90.88%. The general characteristics of the 1475 surveyed subjects were summarized in Table 1: male students accounted for less than a half (45.08%); a predominant majority of students were undergraduates (73.22%); 31.53% majored in agriculture or forestry. All surveyed students reported a high level of attitude or willingness to practice in biodiversity conservation: 68.68% deemed biodiversity conservation as “very important”, and 84.88% were willing to participate in biodiversity conservation activities that were around them.

**Table 1:** General characteristics of study subjects (*N* = 1475).

Characteristics	*N* (%)/Median (Inter-quartiles-range, IQR)
Sex: Male	665 (45.08)
Race: Han majority	1105 (74.92)
Single child: Yes	351 (23.80)
Education level of father	
Primary school and below	548 (37.15)
Junior high school	620 (42.03)
Senior high school and above	307 (20.81)
Education level of mother	
Primary school and below	742 (50.31)
Junior high school	494 (33.49)
Senior high school and above	239 (16.20)
Family residence	
Village	1037 (70.31)
Township or city	438 (29.69)
Student type	
Pre-college students	279 (18.92)
Undergraduates	1080 (73.22)
Postgraduates	116 (7.86)
Major	
Agriculture or forestry	465 (31.53)
Other	1010 (68.47)
Average score of courses	
≥80	632 (42.85)
<80	843 (57.15)
Social support	
Average score in general	3.95 (0.62)
Average score for parents	3.89 (0.89)
Average score for teachers	4.00 (0.70)
Average score for classmates	4.00 (0.70)
Average score for close friends	4.00 (0.70)
Attitude of biodiversity conservation: very important	1013 (68.68)
Willingness to practice biodiversity conservation: willing to	1252 (84.88)

### 3.2 Associated factors of attitude and willingness to practice in biodiversity conservation

For attitude of biodiversity conservation, multivariate binary logistic regression model revealed that, after adjustment, major, average score of the courses, and average score of social support were prominently associated with attitude to practice in biodiversity conservation: compared with university students who majored in agriculture or forestry, those who majored in other subjects were less likely to perceive that biodiversity conservation is “very important” (Odds ratio, OR = 0.52; 95% confidence interval, CI: 0.39–0.70); students with lower average score in courses (<80) also were less likely to put a higher level of importance on biodiversity conservation (OR = 0.62, 95% CI: 0.49–0.78); a 1 point increase in average social support score was associated with increased likelihood in recognizing the significance of biodiversity conservation (OR = 1.56, 95% CI: 1.35–1.81) (Table 2).

**Table 2:** Univariate and multivariate logistic regression analysis results for associated factors of attitude and willingness to practice biodiversity conservation.

Factors	Attitude (Very important)		Willingness (Willing to)	
	Crude OR (90% CI)	Adjusted OR (95% CI)	Crude OR (90% CI)	Adjusted OR (95% CI)
Sex (Ref: Male): Female	1.19 (0.99, 1.43)		*1*.*33 (1*.*05*, *1*.*69)*	1.24 (0.91, 1.67)
Race (Ref: Han majority): Minorities	*1*.*25 (1*.*00*, *1*.*55)*	1.25 (0.96, 1.65)	*1*.*48 (1*.*10*, *2*.*01)*	1.30 (0.91, 1.91)
Single child (Ref: Yes): No	0.90 (0.72, 1.12)		1.25 (0.95, 1.63)	
Education level of father (Ref: Primary school and below)				
Junior high school	0.90 (0.73, 1.11)	0.78 (0.59, 1.03)	0.80 (0.61, 1.05)	
Senior high school and above	*0*.*68 (0*.*53*, *0*.*87)*	*0*.*60 (0*.*41*, *0*.*88)*	0.79 (0.57, 1.10)	
Education level of mother (Ref: Primary school and below)				
Junior high school	*1*.*24 (1*.*00*, *1*.*53)*	*1*.*47 (1*.*10*, *1*.*95)*	0.79 (0.60, 1.04)	0.73 (0.51, 1.03)
Senior high school and above	0.85 (0.65, 1.09)	1.08 (0.74, 1.60)	*0*.*61 (0*.*45*, *0*.*85)*	*0*.*63 (0*.*40*, *0*.*99)*
Family residence (Ref: Village): Township or city	0.92 (0.75, 1.12)		*0*.*77 (0*.*60*, *0*.*99)*	0.89 (0.63, 1.28)
Student type (Ref: Pre-college students)				
Undergraduates	0.93 (0.73, 1.18)	0.83 (0.61, 1.13)	0.92 (0.67, 1.25)	
Postgraduates	*2*.*05 (1*.*32*, *3*.*26)*	1.21 (0.68, 2.23)	1.49 (0.86, 2.72)	
Major (Ref: Agriculture or forestry): Other	*0*.*50 (0*.*40*, *0*.*62)*	*0*.*52 (0*.*39*, *0*.*70)*	*0*.*49 (0*.*36*, *0*.*65)*	*0*.*63 (0*.*43*, *0*.*90)*
Average score of courses (Ref: ≥80): <80	*0*.*60 (0*.*49*, *0*.*72)*	*0*.*62 (0*.*49*, *0*.*78)*	0.95 (0.74, 1.20)	
Average score of social support: +1	*1*.*61 (1*.*43*, *1*.*81)*	*1*.*56 (1*.*35*, *1*.*81)*	*1*.*63 (1*.*42*, *1*.*86)*	*1*.*47 (1*.*23*, *1*.*75)*
Attitude (Ref: Important and below): Very important	_*NA*	*NA*	*4*.*34 (3*.*39*, *5*.*58)*	*3*.*73 (2*.*75*, *5*.*08)*

For willingness to practice in biodiversity conservation, major, average score of social support, and attitude of biodiversity conservation were significant associated factors: compared with students majored in agriculture or forestry, those who majored in other subjects were also less likely to report willingness (OR = 0.63, 95% CI: 0.43–0.90); a 1 point increase in average social support score was associated with higher likelihood of willingness (OR = 1.47, 95% CI: 1.23–1.75); students who recognized biodiversity conservation as “very important” were more likely to report willingness (OR = 3.73, 95% CI: 2.75–5.08) (Table 2).

### 3.3 Mediation of social support in the association between attitude and willingness

**Fig 1:**
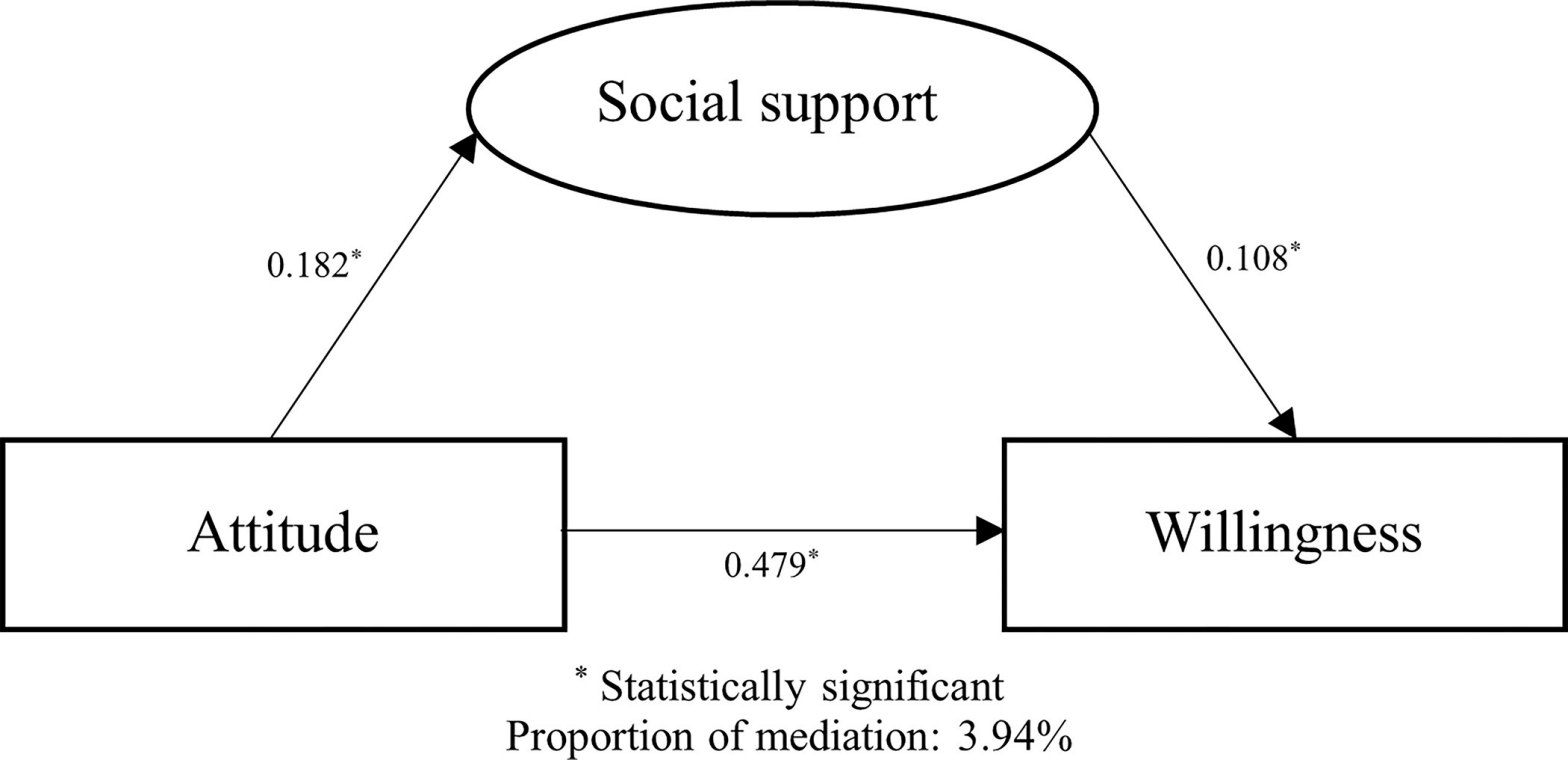
Mediation of social support in the association between attitude and willingness of biodiversity conservation.

**Fig 2:**
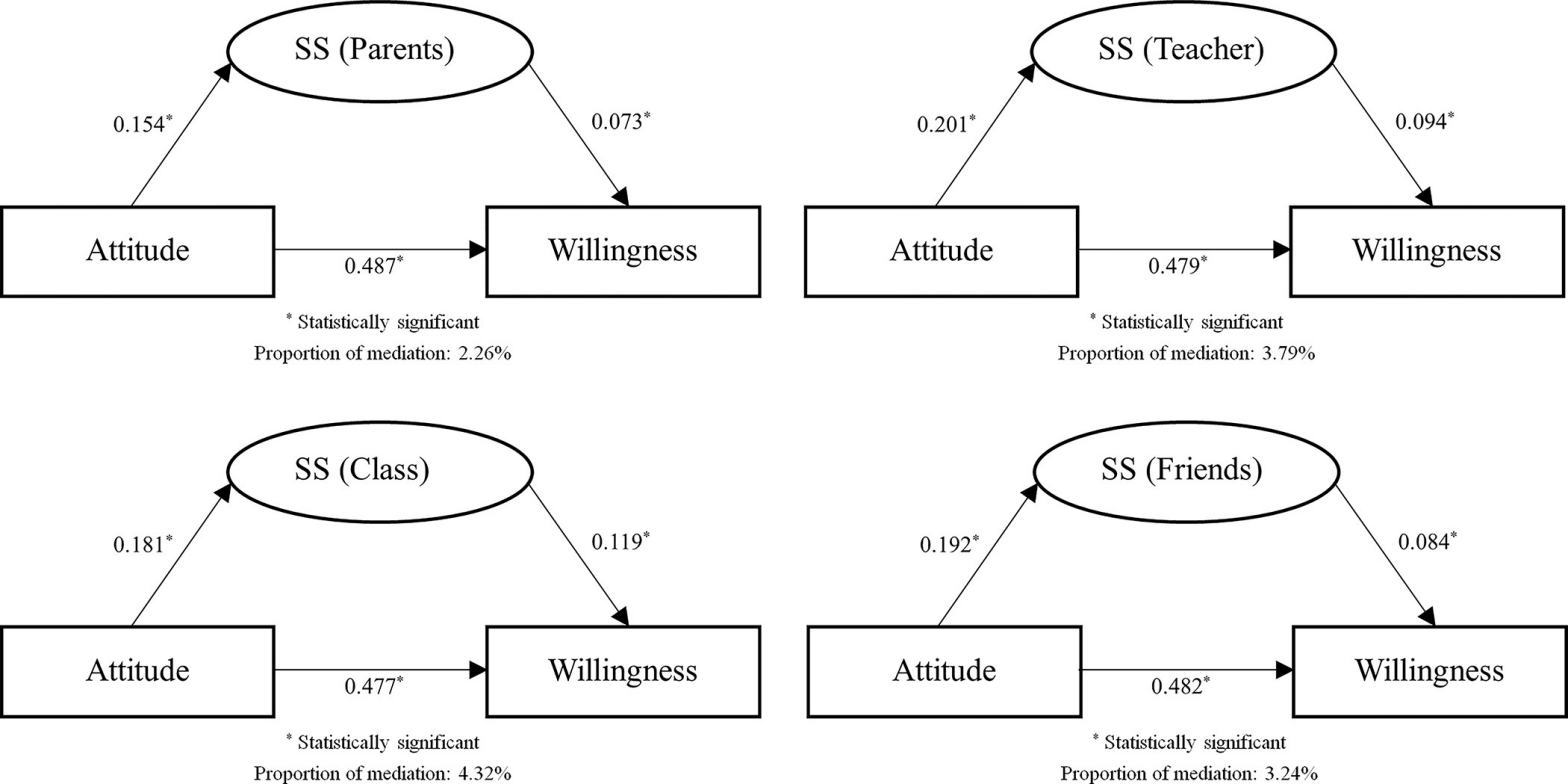
Mediation of different types of social support (SS) in the association between attitude and willingness of biodiversity conservation.

We performed path analysis to evaluate the mediation of social support in the association between attitude and willingness of biodiversity conservation. The analytical results revealed that social support in general presented statistically significant mediation in this association, accounted for 3.94% of the total association (Fig 1). As for different types of social support, social support from classmates mediated the largest proportion of the association (4.32%), followed by social support from teachers (3.79%), from friends (3.24%), and from parents (2.26%) (Fig 2). A series of stratified analyses were further done, and the results were jointly summarized in Table 3: sex, race, and residence exhibited prominent effect modification on mediation of social support, the mediation was only statistically significant in male, Han majority, and students whose family residences located in townships or cities.

**Table 3:** Proportion of mediation for different types of social support (SS) in the association between attitude and willingness of biodiversity conservation by different stratification factors.

Stratified by		SS (Total)	SS (Parents)	SS (Teacher)	SS (Class)	SS (Friends)
Sex	Male	5.92%	3.95%	5.52%	6.92%	4.65%
	Female	0%	0%	0%	0%	0%
Race	Han majority	5.17%	3.00%	4.48%	5.45%	4.78%
	Minorities	0%	0%	0%	0%	0%
Residence	Village	0%	0%	0%	0%	0%
	Township or city	6.29%	3.57%	6.18%	6.28%	5.64%
Major	Agriculture or forestry	3.59%	0%	4.24%	4.19%	0%
	Other	4.21%	1.87%	3.78%	4.55%	4.60%

## 4 Discussion

In this cross-sectional study of 1475 Chinese university students, we explored associated factors of attitude and willingness in biodiversity conservation, we found that major and social support were statistically associated with both attitude and willingness of biodiversity conservation. Besides, a prominent relationship between attitude and willingness of biodiversity conservation has been identified, and social support presented as significant mediating factor in this relationship. Moreover, sex, race, and family residence significantly modified the proportion of mediation by social support. These major findings provide useful information in constructing targeted intervention measures which aiming at promoting biodiversity conservation action in Chinese university students.

Although the university students that we have surveyed in this study reported generally high level of biodiversity conservation attitude and willingness, analytical results suggest some subpopulations should be focused on for further improvements. For instance, compared with students who majored in forestry or agriculture, students of other majors reported much lower attitude and willingness in biodiversity conservation. In recent years, the concept of “green education”, which integrates environmental education, environmental protection, and sustainable development, has been emphasized and promoted by the Chinese government at different levels of schools, from kindergarten to university [16], which has led to some progress at both the theoretical and practical levels. However, a newly published study revealed that for many universities, the “green education” has been implemented only occasionally and superficially, and this passive implementation can easily result in loss of motivation in learning [17]. Our findings corroborate that, at the national level, curriculum relates to biodiversity conservation should be greatly strengthened in Chinese universities, particularly for students who majored outside biology related sciences.

As expected, we found that social support played an important role in biodiversity conservation of Chinese university students, with increased social support associated with higher level of either attitude or willingness of biodiversity conservation. Although the influence of social support on KAP of sustainability has seldom been discussed in existing literature, numerous studies have been published in supporting the significant connection between social support and attitude and practice of other behaviors. For instance, a higher level of social support was associated with more positive attitudes towards death in older patients on hemodialysis [18], more positive the teachers’ attitudes toward inclusive education [19]. Aside from this direct influence, we also found that social support presented as a significant mediator in the association between attitude and willingness of biodiversity conservation, especially for social support from classmates and teachers. All the above findings suggest that, for promoting Chinese university students’ attitude and willingness toward biodiversity conservation, intervention measures which aiming at consolidating social support, particularly social support from classmates and teachers, could be considered. Currently, some effective methods have been proven effective in enhancing social support. In a 2019 randomized controlled trial of 60 Chinese university students, the researchers found that group-based interpersonal psychotherapy, a structured, attachment-focused psychotherapy, significantly improved social support of the study subjects, and the effect was lasting [20].

Another important finding of our study would be that some demographic factors, like sex, race, and residence, showed prominent effect modification on the mediation of social support in the association between attitude and willingness of biodiversity conservation. When performing stratified analysis by these factors, the mediation of social support was only statistically significant in male, Han majority, and students who resided in townships or cities. Compared with ethnic minorities and students who resided in villages, those who were Han majority or resided in townships are generally in higher level of socioeconomic status (SES) [21]. Therefore, our findings may suggest that SES could be a prominent effect modifier in mediation of social support. Two previously published studies provided indirect evidence on this assumption as they identified that SES was significant moderator in the association between social support and health-related outcomes [22, 23]. However, why the mediation of social support is only significant in male students should be further investigated. Under any circumstance, these findings highlight subpopulations that should be focused on in boosting biodiversity conservation attitude and practice among Chinese university students.

Although our study is by far the largest cross-sectional study in investigating attitude and willingness of biodiversity conservation in Chinese university students, several limitations should be acknowledged. First, although we adopted probabilistic sampling method to choose study subjects, as all participants were students from a single university, the representativeness of our study sample to all Chinese university students is questionable. Second, because of the cross-sectional nature, the causal inference based on our study results cannot be reached. Future studies of more representative sample of Chinese students and longitudinal design should be done, to further corroborate our major findings.

## Supporting information

S1 Data (DOCX)
