# Supplementary material for: Attitude and willingness of biodiversity conservation in Chinese university students: Associated factors and the mediation of social support
Source: PLoS One. 2024 Jul 19;19(7):e0307510. doi: 10.1371/journal.pone.0307510 (PMC11259281; doi:10.1371/journal.pone.0307510)
Supplement: S1 Data — (DOCX) [file pone.0307510.s001.docx]

setwd("D:/Normal")

library(readxl)

library(dplyr)

library(haven)

library(psych)

library(gmodels)

data<-read_excel("database.xlsx")

CrossTable(data$b32)

#####recode several variables#############

data$faedu[data$a6<=2]<-1

data$faedu[data$a6==3]<-2

data$faedu[data$a6>=4]<-3

data$moedu[data$a7<=2]<-1

data$moedu[data$a7==3]<-2

data$moedu[data$a7>=4]<-3

data$place[data$a10==1]<-1

data$place[data$a10>=2]<-2

data$grade[data$a12<=2]<-1

data$grade[data$a12>=3 & data$a12<=6]<-2

data$grade[data$a12>=7]<-3

data$major[data$a15==3]<-1

data$major[data$a15<=2 | data$a15>3]<-2

data$score[data$a17<=2]<-1

data$score[data$a17>=3]<-2

data$taidu[data$b21<=3]<-0

data$taidu[data$b21>=4]<-1

data$yiyuan[data$b32<=3]<-0

data$yiyuan[data$b32>=4]<-1

############Social support score#############

data$ssparent<-data$a191+data$a192+data$a193+data$a194+data$a195+

+data$a196+data$a197+data$a198+data$a199

data$ssparent2<-data$ssparent/9

data$sste<-data$a1910+data$a1911+data$a1912+data$a1913+data$a1914+

+data$a1915+data$a1916+data$a1917+data$a1918+data$a1919

data$sste2<-data$sste/10

data$ssclass<-data$a1920+data$a1921+data$a1922+data$a1923+data$a1924+

+data$a1925+data$a1926+data$a1927+data$a1928+data$a1929

data$ssclass2<-data$ssclass/10

data$ssfri<-data$a1930+data$a1931+data$a1932+data$a1933+data$a1934+

+data$a1935+data$a1936+data$a1937+data$a1938+data$a1939

data$ssfri2<-data$ssfri/10

data$sstotal<-(data$ssparent+data$sste+data$ssclass+data$ssfri)/39

describe(data$sstotal)

##Univariate and multivariate analysis for attitude and willingness######

data$a1<-factor(data$a1)

data$a2<-factor(data$a2)

data$a3<-factor(data$a3)

data$faedu<-factor(data$faedu)

data$moedu<-factor(data$moedu)

data$place<-factor(data$place)

data$grade<-factor(data$grade)

data$major<-factor(data$major)

data$score<-factor(data$score)

CrossTable(data$a2,digits=4)

#####attitude#######

fit<-glm(taidu~major,data=data,family="binomial")

summary(fit)

confint(fit,level=0.90)

fit2<-glm(taidu~a2+faedu+moedu+grade+major+score+sstotal, data=data, family="binomial")

summary(fit2)

confint(fit2,level=0.95)

####willingness#####

fit<-glm(yiyuan~taidu,data=data,family="binomial")

summary(fit)

confint(fit,level=0.90)

fit2<-glm(yiyuan~a1+a2+moedu+place+major+sstotal+taidu,data=data,family="binomial")

summary(fit2)

confint(fit2,level=0.95)

########Associations between attitude and SS########

model1<-lm(sstotal~a1+a3+faedu+moedu+major+score+taidu,data=data)

summary(model1)

##########path analysis#########

library(lavaan)

model1<-'

b21~faedu+major+score

ssfri2~a1+faedu+moedu+major+score+b21

b32~b21+ssfri2+a1+moedu+major

'

results<-cfa(model1,data=data,ordered=c("b21","b32","faedu","moedu"))

summary(results,fit.measures=TRUE,standardized=T,rsquare=T)

######subgroup analysis###########

library(lavaan)

sub1<-subset(data,data$major==2)

model1<-'

b21~faedu+score

ssfri2~faedu+moedu+score+b21

b32~b21+ssfri2+moedu

'

results<-cfa(model1,data=sub1,ordered=c("b21","b32","faedu","moedu"))

summary(results,fit.measures=TRUE,standardized=T,rsquare=T)
